# *Astragalus membranaceus* Modulates Inflammatory Markers Without Enhancing Muscle Function Following Intensified Resistance Training

**DOI:** 10.3390/nu18101598

**Published:** 2026-05-18

**Authors:** Simone Villanova, Marco Gatti, Marta Colosio, Letizia Giusti, Giulia Papetti, Pietro Blumetti, Simone Porcelli

**Affiliations:** 1 Department of Molecular Medicine, University of Pavia, 27100 Pavia, Italy; s.villanova@studenti.uniroma4.it (S.V.); marco.gatti03@universitadipavia.it (M.G.); colosio@wisc.edu (M.C.); letizia.giusti01@universitadipavia.it (L.G.);; 2 Department of Movement, Human and Health Sciences, University of Rome ‘Foro Italico’, 00135 Rome, Italy; 3 Department of Medicine, Division of Geriatrics & Gerontology, University of Wisconsin–Madison, Madison, WI 53705, USA; 4 Medical Center srl, Sesto Calende, 21018 Varese, Italy; pietro@pblumetti.com; 5 IRCCS Fondazione Policlinico San Matteo, 27100 Pavia, Italy

**Keywords:** non-functional over-reaching, dietary supplementation, inflammation, muscle damage

## Abstract

**Background:** Astragali radix is a traditional herb known for its antioxidant, anti-inflammatory, and immunomodulatory properties and has gained attention for its potential to support post-exercise recovery. However, the effects of long-term supplementation coupled with resistance training are not well understood. **Methods:** Twenty-four moderately active participants were recruited and randomly assigned to the Astragali radix supplementation (ASTRA, *n* = 13) or placebo (PLA, *n* = 11) group. All participants underwent 8 weeks of regular resistance training (3 sessions/week) and 2 weeks of intensified training (6 sessions/week). **Results:** Before (BAS), after 8 weeks of resistance training (RT), and at the end of the intensified training (IT), knee extensors’ maximal voluntary isometric contraction torque (MVIT), and leg press and leg extension one repetition max (1RM) were measured. Blood samples were collected to analyze inflammatory markers and testosterone. From BAS to after RT, MVIT, 1RM leg press, and 1RM leg extension increased in both ASTRA and PLA, with no differences between groups. After IT, MVIT, 1RM leg press and 1RM leg extension decreased in both ASTRA and PLA. CPK levels and myoglobin concentration increased while cortisol decreased significantly from BAS to IT, but no group differences were detected. TNF-α and IL-6 showed significant time × supplementation interactions, with lower values after IT in ASTRA compared to PLA. **Conclusions:** Astragali radix supplementation did not lead to additional benefits in muscle during the period of resistance training, nor did it prevent the decline in force following the intensified training period. However, Astragali radix supplementation prevented the increase in some inflammatory biomarkers, specifically TNF-α and IL-6, during the intensified period of training.

## 1. Introduction

Physical inactivity accounts for approximately 10% of premature deaths and an estimated €100 billion in annual healthcare costs [1]. Current guidelines recommend at least 150 min of moderate-intensity aerobic activity per week, with additional resistance training sessions to improve bone mineral density, cardiovascular risk, and metabolic health [2,3,4]. For individuals engaged in chronic training programs, however, an imbalance between training load and recovery can lead to non-functional overreaching (NFOR) or, in more severe cases, to overtraining syndrome (OTS) [5,6]. At the skeletal muscle level, NFOR has been associated with impaired beta-2 adrenergic signaling and altered mitogen-activated protein kinase activity [5,7], alongside hormonal dysregulation, including reduced testosterone and an altered cortisol/testosterone ratio [8], and elevated muscle damage biomarkers such as creatine kinase [9,10].

Recent studies have indicated that certain dietary interventions, such as a carbohydrate-rich diet, can help prevent the onset of OTS and facilitate recovery [11]. Moreover, a recent review by Tanabe et al. [12] summarized the effects of various dietary supplement strategies on mitigating the undesired consequences of exercise-induced impairments on muscle functional properties, muscle damage, and joint function. Positive effects have been demonstrated with curcumin, tart cherry juice, beetroot juice, and quercetin due to their involvement in the inflammatory cascade triggered by muscle damage and their antioxidant properties [12]. However, findings across supplements remain heterogeneous, and their effectiveness during prolonged periods of intensified resistance training is still unclear.

Among the plethora of various dietary supplements, Astragali radix, one of the most well-known herbs in Chinese traditional medicine and recently included in the European Pharmacopoeia (8th ed., EDQM, Council of Europe), has garnered attention due to its antioxidant, anti-inflammatory, and immunomodulatory properties [13,14]. Research on Astragali radix extract supplementation has primarily focused on clinical populations, including cancer patients, and has demonstrated positive effects as supportive care in reducing inflammation and enhancing immune function [15]. More recently, studies involving healthy individuals suggested that Astragali radix extract supplementation might have a protective effect by reducing markers of damage and inflammation [16]. For instance, Yeh and colleagues demonstrated that levels of creatine kinase (CPK) and lactate dehydrogenase (LDH), two common enzymes used as biomarkers of muscle damage, were reduced, and muscle force production was less impaired after an eccentric exercise bout [17]. Additionally, Chen and colleagues found that 8 weeks of Astragali radix supplementation enhanced aerobic performance in runners compared to a placebo group [18]. Another recent study reported that after 28 days of Astragali radix extract supplementation (480 mg), joint pain was significantly reduced in 45 adults with functional knee joint pain (not linked to acute injury or chronic disease) [19]. Furthermore, a recent comprehensive review by Lin et al. [20] highlighted the molecular mechanisms underlying the protective effects of Astragali radix, including its role in modulating oxidative stress, regulating cell death pathways, and enhancing immune responses.

Despite emerging evidence, the current literature remains limited by the small number of studies conducted in athletic populations, the heterogeneity of outcome measures, and the predominance of short-term, acute interventions. The potential effectiveness of Astragali radix supplementation during a prolonged period of intensified resistance training, a condition that often challenges recovery capacity, remains unknown. Moreover, no previous investigation has specifically examined supplementation during a structured resistance training program including a period of intensified loading.

Therefore, the present project aims to evaluate whether Astragali radix extract supplementation can have protective effects on joint pain, muscle function, and exercise-induced muscle damage in recreational athletes undergoing a 10-week resistance training period (8 weeks of regular resistance training followed by 2 weeks of intensified training). This is the first study to evaluate prolonged Astragali radix supplementation during a structured intensified resistance training protocol in recreational athletes, a condition that challenges recovery capacity and has not previously been investigated with this supplement. We hypothesize that Astragali radix extract supplementation will mitigate the negative effects of an intensified training intervention on muscle force, joint pain, and perceived fatigue after 10 weeks of resistance training.

## 2. Methods

### 2.1. Participants

**Figure 1:**
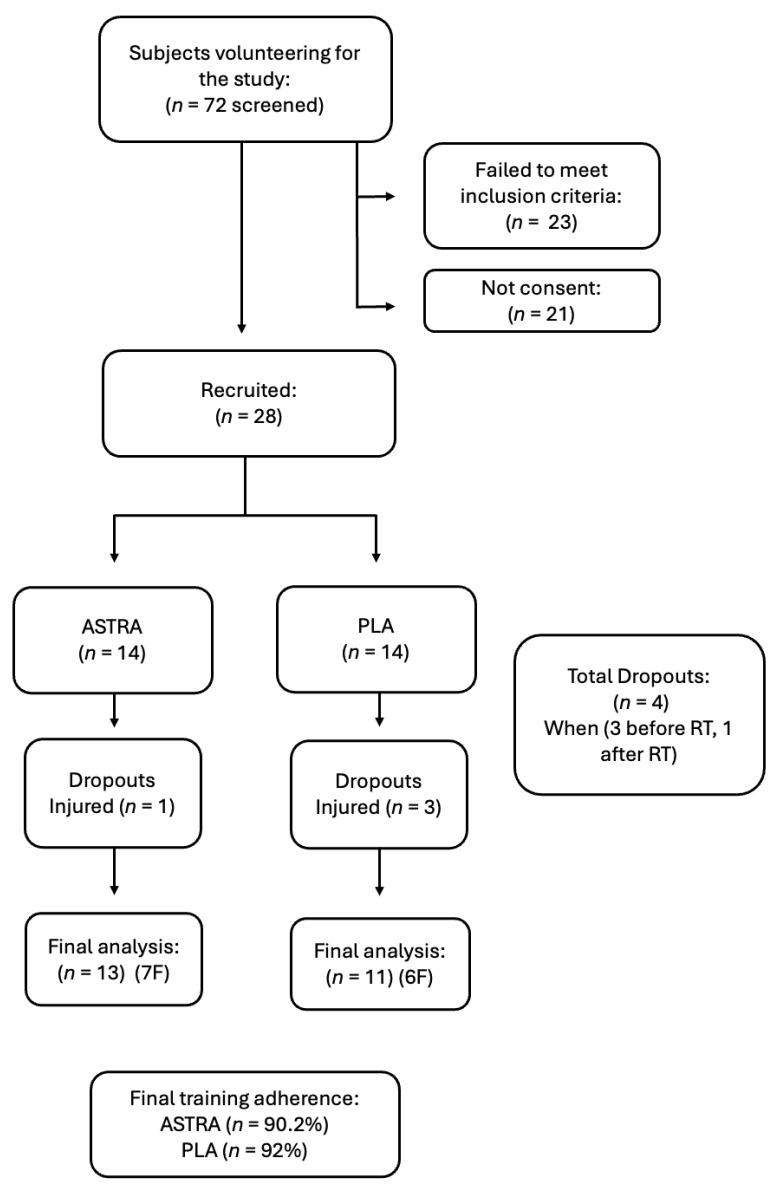
Flow diagram illustrating the process of subject screening, selection, and enrollment for study participation. Participants were allocated to either the placebo group (PLA) or the Astragali radix extract supplement group (ASTRA).

Participants were selected from a large population of students attending courses at the University of Pavia, Italy. The participants had to meet the following criteria to be recruited: (i) non-smokers; (ii) moderately active (between 3 and 6 times the metabolic equivalent of the resting oxygen consumption (MET)) based on the International Physical Activity Questionnaire—Short Form (IPAQ-SF) [21]; (iii) free from muscular, bone, or joint injuries in the previous 6 months; and (iv) free from cardiovascular, metabolic, and neurological diseases. Students with a history of taking dietary supplements or drugs in the 6 months before the start of the study were also excluded. After the initial screening of seventy-two (*n* = 72) volunteers, twenty-eight participants met the inclusion criteria and were enrolled in the project. However, four participants (*n* = 4) dropped out of the study, resulting in a final sample size of twenty-four participants (*n* = 24), who were included in the data analysis (Figure 1). Of the twenty-four participants in the study, blood sampling was performed in twenty-two participants (*n* = 22) since two participants declined to consent to this procedure. All procedures conformed to the Declaration of Helsinki and were approved by the local Ethics Committee (prot. 0040463/23 UNIPV08/08/2023). The students were fully informed of the procedures and possible risks associated with the experiments before they gave written consent to participate in the study. The trial was registered on clinicaltrials.gov (NCT07552675).

### 2.2. Experimental Design

The study used a randomized, double-blind, placebo (PLA)-controlled design. Randomization was performed using a Microsoft Excel spreadsheet. Each participant was assigned a randomly generated number using the “RAND(*x*)” function, after which, the values were ranked in ascending order. Based on this ranking, half of participants were allocated to the ASTRA group and the remaining half to the PLA group. All participants performed 8 weeks of resistance training (RT) and 2 weeks of intensified training (IT). Thirteen participants (*n* = 13; 7 females and 6 males; age: 25 ± 3 years; height: 172 ± 6 cm; weight: 67 ± 12 kg) received, in accordance with the manufacturer’s instructions, 480 mg daily of Astragali radix extract (ASTRA) for 10 weeks (2 capsules per day orally); eleven participants (*n* = 11; 6 females and 5 males; age: 25 ± 4 years; height: 170 ± 7 cm; weight: 67 ± 11 kg) received 2 capsules per day containing a combinations of inert substances with a negligible content of Astragali radix (PLA) for the same period. The two supplements were matched in flavor, appearance, and packaging. Tests were performed one week before the start of the intervention (BAS), 48 h after the RT phase, and 24 h following the completion of the IT phase. The participants were instructed to ingest the final capsule of Astragali radix extract or the placebo 24 h before the scheduled test. All the testing sessions were conducted in a well-ventilated laboratory at 19–21 °C. The participants were instructed to arrive at the laboratory rested and fully hydrated, avoiding strenuous exercise in the 24 h preceding each testing session. They were also asked to avoid alcohol and caffeine products 48 h before the exercise test. Each participant completed one familiarization session 10 days before BAS, which consisted of submaximal and maximal isometric contractions of knee extensors and range of motion measurements.

### 2.3. Training Intervention

A general overview of the training program is shown in Table 1. During RT, the participants performed three sessions of strength training per week for 8 weeks, targeting the lower limb muscles. In each visit, the participants performed three sets, 8–12 repetitions each, of the following exercises: back squat, leg press, single-leg extensions, single-leg curl, and calf raises. The workload was progressively increased from 70% to 80% of 1 repetition max (1RM). To optimize training adaptations and ensure individualized load prescription throughout the intervention, 1RM assessments were performed at BAS, after 4 weeks, and after RT. During IT, the participants completed six sessions per week (12 training sessions total), performing 10 sets, 1 repetition each, at 100% 1RM of leg press and leg extension. If any attempt for the subsequent set was unsuccessful, the workload was decreased by 5 kg to allow the participants to lift as close as possible to their maximum. This protocol has been previously proposed in the literature and is capable of inducing an effective decline in the level of strength [7]. All training sessions were carefully supervised by highly experienced personnel with extensive expertise in strength and conditioning.

### 2.4. Supplementation

Astragali radix supplement and placebo were provided by Giellepi S.p.A. (Seregno, MB, Italy). The active ingredient used in this study, Axtragyl^®^, is a natural extract from the roots of *Astragalus membranaceus*. It was incorporated into the capsules at a concentration of 240 mg per capsule. Each capsule formulation included cellulose (110 mg) as a filler agent and hydroxypropylmethylcellulose (95 mg) as a capsule coating agent. The selected dose (480 mg/day) was based on previous human studies demonstrating beneficial effects at comparable dosages [19,22]. The PLA group received capsules containing an inert substance identical in terms of visual appearance, taste, and smell to the active formulation but without any active ingredients (microcrystalline cellulose (E460i) (173.25 mg), maltodextrin (173.25 mg), and magnesium salts of fatty acids (3.50 mg) encapsulated in hydroxypropyl methylcellulose capsules). The participants were instructed to take two capsules daily at the same time (around 10:00 am and 4:00 pm). The investigational product (Axtragyl) is a hydroalcoholic extract obtained from a membranaceus root, standardized based on the main phytochemical components (including terpenes, flavonoids, and typical astragalus polysaccharides) found in the plant. Participant adherence to the supplementation protocol was actively monitored through daily text messages, and compliance was further confirmed by collection of the returned empty containers at the end of the intervention.

### 2.5. Maximal Voluntary Isometric Torque

Maximal voluntary isometric torque (MVIT) was measured on a custom-built ergometer [23]. The participants were seated and secured by chest and hip straps, with a hip angle of 110° and a knee angle of 90° (as 180°of full extension). Knee extensor force was measured using a calibrated force transducer (SML load cell, Interface Inc., Scottsdale, AZ, USA) attached via a non-compliant strap to the dominant leg (all participants were right leg dominant, as reported using the revised Waterloo Footedness Questionnaire) [24], immediately proximal to the malleoli of the ankle joint. Force was collected at a sampling rate of 2048 Hz, analog-to-digital converted (Load Cell Adapter, Delsys INC, Natick, MA, USA; bandwidth: DC–50 Hz) and transferred via Wi-Fi to the acquisition system (Trigno Base Station, Delsys INC, Natick, MA, USA) connected to a computer. Torque (N·m) was calculated as the force measured by the load cell multiplied by the length of the lever arm and baseline-corrected prior to analysis. After an adequate warm-up consisting of sub-maximal knee extensor contractions, the participants were instructed to “push as hard and fast as possible” for 4 s. The MVIT was considered the greatest force produced over a 250 ms time interval. The participants performed three trials, each separated by 60 s of rest. Visual feedback of the instantaneous force and verbal encouragement were provided during contractions. All data were analyzed offline using Labchart (Labchart software v8, ADInstrument, Oxford, UK).

### 2.6. Range of Motion

The range of motion (ROM) of the knee was calculated during a passive movement of the dominant lower limb [25]. The participant was seated on the examination bed while an experienced operator passively moved the participant’s leg from the zero-reference position (180°, full extension) through the full range of knee flexion until the participant reported the first sensation of discomfort. To ensure consistency, a single experienced operator passively moved the dominant leg of all participants. Each passive movement was repeated 10 times, and the average of the best three trials was considered for the analysis with a variability across all 10 repetitions of less than 5%. ROM was quantified using a gyroscope (Trigno Avanti Sensor, Delsys INC, Natick, MA, USA) fixed at the ankle and data were transferred via Wi-Fi to the acquisition system (Trigno Base Station, Delsys INC, Natick, MA, USA) connected to a computer. To calculate the degree of movement, the integral of the speed curve was calculated from the starting point to the return to baseline. This gyroscope-based approach, based on angular velocity integration, is consistent with previously described IMU-based methodologies for joint kinematic assessment, where gyroscope-derived angular velocity is commonly used to estimate joint motion during dynamic tasks and provides high within-session consistency for within-subject comparisons [26,27]. A similar approach is widely used in orthopedic settings for ROM assessment [28].

### 2.7. Muscle Soreness

Muscle soreness was evaluated using a visual analog scale (VAS) with a 100 mm line that had “no pain” at one end and “extremely sore” at the other end [29]. The subjects were asked to mark their pain level on the line under the supervision of the examiner while the knee joint was passively flexed and extended by the investigator. The same experienced operator performed all assessments with standardized speed and force.

### 2.8. Blood Sample

The blood samples were taken at each time point (BAS, RT, and IT phases) between 7:30 and 8:30 AM to minimize diurnal variations in biomarker levels. Specifically, blood collection was performed 48 h after the last training session of the RT phase and 24 h after the IT phase. Blood collection was performed on the same days as the other assessments and within the same experimental session under standardized conditions. Resting venous blood (~13 mL) was collected from the antecubital vein with the participant in a supine position on the examination bed. The blood samples were drawn into vacuum tubes containing either EDTA or Silica Clot Activator (BD Vacutainer, Dickinson and Company, Franklin Lakes, NJ, USA). Specifically, the EDTA tubes used were BD Vacutainer, EDTA K2E 7.2 mg, and the Clot Activator tubes were BD Vacutainer SST II Advance. Serum creatine phosphokinase (CPK), C reactive protein (CRP), lactate dehydrogenase (LDH), and myoglobin (Mb) levels were assessed using a DT-60 II analyzer (Johnson & Johnson, Rochester, NY, USA). Insulin-like growth factor-1 (IGF-1), interleukin-6 (IL-6) and tumor necrosis factor-alpha (TNF-α) levels were analyzed using enzyme-linked immunosorbent assays (ELISAs) (R&D Systems, Minneapolis, MN, USA). Cortisol and testosterone assays were performed using radioimmunoassay and reagent kits from Farmos Diagnostics (Turku and Oulunsalo, Finland).

### 2.9. Statistical Analysis

According to a statistical power calculation conducted using G*Power (version 3.1), based on changes in MVIT after resistance training as a benchmark, a sample size of 20 subjects (10 per group) was required to achieve a statistical power (beta) of 0.80 and a significance level (alpha) of 0.05. All data are presented as means ± standard deviations (SDs).

A general linear model for repeated measurements with time (BAS, RT, and IT phases) as the within-subject factor and supplementation (ASTRA and PLA) as the between-subject factor was used to evaluate changes in muscle force (MVIT and 1RM), muscle soreness (VAS), joint function (ROM), as well as blood biomarkers (CPK, CRP, LDH, Mb, IGF-1, IL-6, TNF-α, cortisol, and testosterone). The normality of the data was assessed using the Shapiro–Wilk test. The sphericity was checked using Mauchly’s test. When the assumption of sphericity was violated, the significance of *F* ratios was adjusted using Greenhouse–Geisser correction. When significant interactions were observed, Bonferroni’s test was adopted as a post hoc analysis method. As a measure of effect size, partial eta squared (η^2^) was calculated. Statistical significance was set at *p* < 0.05. Statistical analyses were conducted using SPSS software (v. 30.0, IBM, Armonk, New York, USA).

## 3. Results

There were no significant differences between the ASTRA and PLA groups at BAS in any of the measured variables (Table 2).

### 3.1. Training

Participants completed 21 ± 1 of the 24 training sessions prescribed in the RT phase, corresponding to a rate of approximately 88%. During the IT period, 11 ± 0.5 (around 92%) out of 12 strength training sessions were completed.

### 3.2. Muscle Strength (MVIT and 1RM)

**Figure 2:**
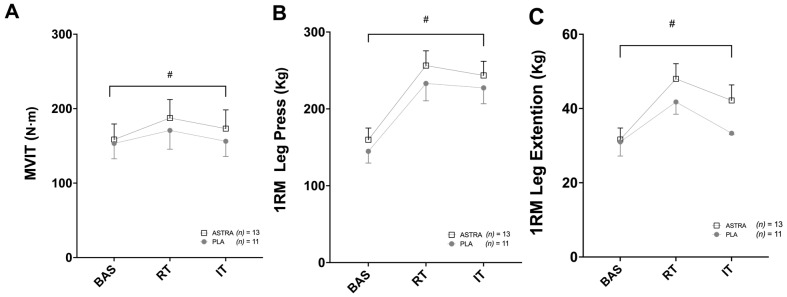
Effect of Astragali radix extract supplementation on (**A**) knee extensor maximal voluntary isometric torque (MVIT), (**B**) maximal repetition (1RM) of leg press, and (**C**) maximal repetition of leg extension after 8 weeks of regular resistance training (RT) and after two weeks of intensified training (IT). Data are presented as a mean ± SD. Placebo group (PLA), Gray circle, (*n*) = 11; Astragali radix extract supplement group (ASTRA), white square, (*n*) = 13; # Time Effect.

MVIT showed a time effect (F _(2,44)_ = 8; *p* = 0.002; ηp^2^ = 0.273) but not a supplementation effect (F _(1,22)_ = 0.1; *p* = 0.692; ηp^2^ = 0.007) or time x supplementation interaction effect (F _(2,44)_ = 1; *p* = 0.500; ηp^2^ = 0.030) (Figure 2A). Specifically, MVIT increased significantly in the RT phase (by around 16 ± 14%; *p* < 0.001) and decreased significantly in the IT phase (by around −8 ± 6%; *p* = 0.004) compared to the RT phase, without significant differences between ASTRA and PLA.

Leg press 1RM also showed a time effect (F _(2, 44)_ = 100; *p* < 0.001 ηp^2^ = 0.820) but not a supplementation effect (F _(1,22)_ = 0.5; *p* = 0.476; ηp^2^ = 0.023) or time x supplementation interaction effect (F _(2,44)_ = 0; *p* = 0.822; ηp^2^ = 0.009) (Figure 2B). Leg press 1RM significantly increased in the RT phase (71 ± 55%; *p* < 0.001) and significantly decreased in the IT phase (−6 ± 5%; *p* = 0.008) compared to the RT phase, with no differences between ASTRA and PLA.

Leg extension 1RM showed a time effect (F _(2,44)_ = 17; *p* < 0.001; ηp^2^ = 0.442) but not a supplementation effect (F _(1,22)_ = 1.6; *p* = 0.218) or time x supplementation interaction effect (F _(2,44)_ = 1.7; *p* = 0.196) (Figure 2C). Leg extension 1RM significantly increased in the RT phase by 38 ± 26% (*p* < 0.001) without differences between groups. In the IT phase, there was no significant difference (−12 ± 16%) in 1RM on leg extension compared to the RT phase.

### 3.3. Muscle Soreness (VAS) and Knee Joint Mobility

**Figure 3:**
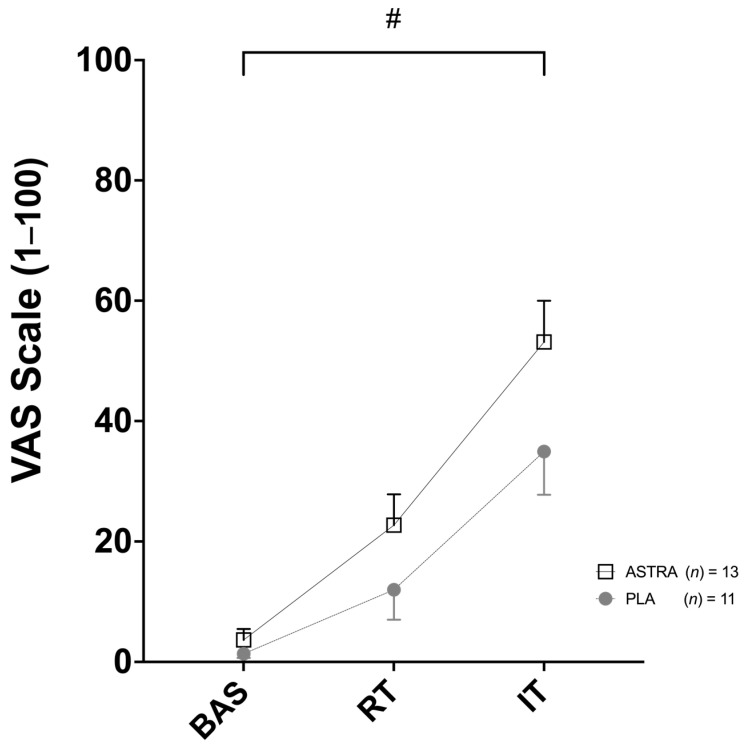
Effect of Astragali radix extract supplementation on muscle soreness measured by visual analog scale (VAS) after 8 weeks of regular resistance training (RT) and after two weeks of intensified training (IT). Data are presented as a mean ± SD. Placebo group (PLA) shown as gray circles (*n* = 11); Astragali radix extract supplement group (ASTRA) shown as white squares (*n* = 13). # time effect (*p* < 0.001).

VAS scores showed a time effect (F _(2,44)_ = 44; *p* < 0.001; ηp^2^ = 0.669) but no supplementation effect (F _(1,22)_ = 4; *p* = 0.052; ηp^2^ = 0.161) or time x supplementation interaction effect (F _(2,44)_ = 1.6; *p* = 0.221; ηp^2^ = 0.066) (Figure 3). Bonferroni post hoc analysis revealed a significant increase after both RT and IT compared to BAS (*p* < 0.001) as well as after IT compared to after RT (*p* < 0.001) with similar patterns observed in both ASTRA and PLA conditions. ROM did not show any significant time (F _(2,44)_ = 0.1; *p* = 0.840; ηp^2^ = 0.008), supplementation (F _(1,22)_ = 0.6; *p* = 0.431; ηp^2^ = 0.028), or time × supplementation interaction effect (F _(2,44)_ = 0.1; *p* = 0.919; ηp^2^ = 0.004) with no meaningful changes observed across conditions (ASTRA vs. PLA: BAS 87 ± 4° vs. 88 ± 4°, RT 87 ± 3° vs. 88 ± 4°, IT 87 ± 7° vs. 89 ± 4°, respectively).

### 3.4. Blood Biomarkers

**Figure 4:**
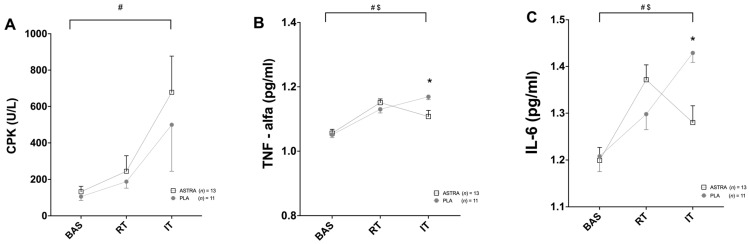
Effect of Astragali radix extract supplementation on blood damage markers: (**A**) serum creatine phosphokinase (CPK), (**B**) tumor necrosis factor-alpha (TNF-α), and (**C**) interleukin-6 (IL-6) levels after 8 weeks of regular resistance training (RT) and after two weeks of intensified training (IT). Data are presented as the mean ± SD. Placebo group (PLA) shown as gray circles (*n* = 11); Astragali radix extract supplement group (ASTRA) shown as white squares (*n* = 13). # time effect, $ time × supplementation interaction effect, * different compared to ASTRA group.

CPK showed a time effect (F _(2,40)_ = 8.1; *p* = 0.001; ηp^2^ = 0.289) but not a supplementation (F _(1,20)_ = 0.4; *p* = 0.514; ηp^2^ = 0.022) or time x supplementation (F _(2,40)_ = 0.2; *p* = 0.743; ηp^2^ = 0.012) effect (Figure 4A). In the RT phase, the levels of CPK significantly increased (*p* = 0.041), with no differences between groups. In the IT phase, CPK significantly increased from BAS (*p* = 0.004), without differences between or within groups. Myoglobin showed a time effect (F _(2,40)_ = 12; *p* < 0.001; ηp^2^ = 0.392) but not a supplementation (F _(1,20)_ = 0.2; *p* = 0.615; ηp^2^ = 0.013) nor time x supplementation effect (F _(2,40)_ = 0.1; *p* = 0.701; ηp^2^ = 0.013). In the RT phase, the levels of myoglobin significantly increased by 22 ± 11% (*p* = 0.042), with no differences between groups. In the IT phase, myoglobin levels significantly increased by 54 ± 15% (*p* = 0.005) compared to BAS, with no differences between groups. LDH showed no time (F _(2,40)_ = 1; *p* = 0.353; ηp^2^ = 0.051), supplementation (F _(1,20)_ = 0.1; *p* = 0.708; ηp^2^ = 0.007), or time x supplementation interaction (F _(2,40)_ = 0.1; *p* = 0.883; ηp^2^ = 0.003) effect.

TNF-α showed (Figure 4B) a time effect (F _(2,40)_ = 36; *p* < 0.001; ηp^2^ = 0.644) and time × supplementation interaction effect (F _(2,40)_ = 6; *p* = 0.002; ηp^2^ = 0.259), with no main effect from supplementation (F _(1,20)_ = 0.9; *p* = 0.343; ηp^2^ = 0.045). Post hoc analysis revealed that TNF-α levels in both groups increased similarly after RT (9 ± 4% and 7 ± 4% for ASTRA and PLA, respectively; both *p* < 0.001). in contrast, in the IT phase, the TNF-α levels decreased in ASTRA (−4 ± 6%) and increased in PLA (4 ± 3%; *p* = 0.012). Similarly, IL-6 (Figure 4C) showed a significant main effect from time (F _(2,40)_ = 23; *p* < 0.001; ηp^2^ = 0.537) and time × supplementation interaction (F _(2,40)_ = 11; *p* < 0.001; ηp^2^ = 0.354), with no main effect from supplementation (F _(1,20)_ = 0.7; *p* = 0.457; ηp^2^ = 0.032). Bonferroni post hoc revealed that after IT, there was a reduction in IL-6 levels in ASTRA (−6 ± 7%) and an increase PLA (+11 ± 8%; *p* = 0.003). IGF-1 showed a time effect (F _(2,40)_ = 16; *p* < 0.001; ηp^2^ = 0.449), but not a supplementation (F _(1,20)_ = 0.3; *p* = 0.579; ηp^2^ = 0.016) or time × supplementation interaction (F _(2,40)_ = 1.3; *p* = 0.273; ηp^2^ = 0.063) effect. Cortisol showed a time effect (F _(2,40)_ = 10; *p* < 0.001; ηp^2^ = 0.338) but not a supplementation (F _(1,20)_ = 4; *p* = 0.063; ηp^2^ = 0.163) or time × supplementation effect (F _(2,40)_ = 1.6; *p* = 0.206; ηp^2^ = 0.076). In the IT phase, cortisol significantly decreased by 23 ± 20% (*p* = 0.040), with no differences between groups. CRP showed no time (F _(2,40)_ = 1; *p* = 0.270; ηp^2^ = 0.063), supplementation (F _(1,20)_ = 0.8; *p* = 0.374; ηp^2^ = 0.040), or time × supplementation interaction (F _(2,40)_ = 1; *p* = 0.342; ηp^2^ = 0.052) effect. Testosterone showed no time (F _(2,40)_ = 0.1; *p* = 0.910; ηp^2^ = 0.005), supplementation (F _(1,20)_ = 0.1; *p* = 0.812; ηp^2^ = 0.003), or time × supplementation interaction (F _(2,40)_ = 0.5; *p* = 0.626; ηp^2^ = 0.023) effect.

## 4. Discussion

The present study investigated whether Astragali radix extract supplementation could influence muscle function, soreness, and inflammatory responses during an 8-week resistance training program followed by 2 weeks of intensified training in healthy individuals. The principal finding is that supplementation selectively attenuated the post-exercise inflammatory response—specifically reducing circulating TNF-α and IL-6 during the intensified training phase—without conferring measurable benefits on muscle strength, force recovery, joint pain, or hormonal profiles. This pattern of results is both scientifically informative and conceptually important: it demonstrates that the anti-inflammatory properties of Astragali radix are biologically active under conditions of prolonged mechanical overload, yet suggests that attenuation of systemic inflammation alone is insufficient to preserve or restore neuromuscular performance.

### 4.1. Astragali Radix Supplementation and Muscle Force

The resistance training intervention over an 8-week period significantly increased 1RM in both the leg press (around +70%) and leg extension (around +38%), along with a 16% improvement in MVIT of the knee extensors. These gains are similar to those reported in studies using the same exercise intervention [30] and they are typically attributed to neural adaptation in the early phase and muscle structural changes over the longer term [31]. However, when examining the effect of Astragali radix supplementation, the results indicated no additional positive effects on muscle strength improvement when compared to resistance training alone. This is consistent with previous studies on *Astragalus membranaceus* that reported no clear relationship between Astragali radix supplementation and enhanced physical strength [32]. Furthermore, when compared with creatine alone, a combination of Astragali radix, ginseng, and creatine supplementation did not show any superior improvements in strength and lean mass [33]. One potential explanation is that the active components of *Astragalus membranaceus* root extract, specifically polysaccharides and saponins, primarily exert their effects through antioxidant, anti-inflammatory, and immunomodulatory effects [13,14], rather than directly influencing muscle protein synthesis or breakdown. This suggests that while Astragali radix may confer several health benefits, these do not appear to extend to meaningful improvements in muscle force.

Following the IT period of 12 sessions over two weeks, 1RM of leg press decreased by 4%, and MVIT significantly decreased by 8% compared to the RT phase. The force declines observed following the IT period are consistent with values previously reported after comparable intensified resistance training protocols [9,34] and are most plausibly attributable to mechanical overloading combined with insufficient inter-session recovery [35]. However, contrary to our hypothesis, Astragali radix extract supplementation did not show any beneficial effect on muscle force following the IT period as it neither accelerated the recovery of muscle force nor mitigated its decline. Similar results were observed for muscle soreness (VAS) where we observed a remarkable increase after the IT period in both groups.

### 4.2. Astragali Radix Supplementation and Blood Biomarkers

In the present study, the 8-week resistance training period produced biomarker changes consistent with an expected pattern for adaptation to progressive mechanical stress. CPK levels and myoglobin concentrations rose significantly in both groups from BAS to after RT, reflecting cumulative muscle fiber stress—a process that precedes hypertrophic adaptation rather than representing pathological damage per se [36,37]. Importantly, these elevations were comparable between groups, confirming that Astragali radix supplementation did not alter the normal muscle damage response during structured progressive training. Similarly, the equivalent increase in TNF-α observed in both groups after RT is consistent with exercise-induced immune activation and confirms that the inflammatory machinery was engaged to a comparable extent in both groups, irrespective of supplementation. Taken together, the BAS-to-after-RT trajectory provides an important baseline for interpreting the divergent biomarker responses that emerged between groups during the subsequent intensified phase. Following the two weeks of IT, our results showed significantly higher levels of CPK in blood samples and revealed higher secretion of the pro-inflammatory cytokines TNF-α and IL-6. These cytokines play key roles in the inflammatory response and may reflect an early stage of muscle regeneration following an overload training period [38,39]. Taken together, these alterations are consistent with a state of accumulated muscle stress and inadequate recovery, which likely underlies the observed decline in force production during the IT period.

In our study, *Astragalus membranaceus* root extract supplementation positively suppressed the secretion of TNF-α and IL-6, thereby reducing inflammation after the IT period. This is in agreement with prior research [17]. Specifically, Yen and colleagues found that after a single bout of five sets of 10 repetitions of unilateral eccentric leg extensions with a load of 120% of 1RM, *Astragalus membranaceus* root extract supplementation effectively reduced IL-6 and TNF-α and facilitated faster restoration of skeletal muscular strength following eccentric exercise-induced muscle damage [17]. One possible explanation for these results relates to the combination of antioxidative effects, including the stimulation of antioxidant enzymes and the reduction in oxygen-free radicals by secondary metabolites from *Astragalus membranaceus* roots [16]. Other proposed mechanisms include the capacity of *Astragalus membranaceus* to clear metabolic byproducts from muscle tissue and to attenuate the inflammatory response [40]. These findings suggest that Astragali radix supplementation may help attenuate systemic inflammatory responses during intensified training, though the broader implications of this effect for performance and adaptation require careful consideration. Notwithstanding these observations, an important conceptual consideration is whether a reduction in systemic inflammatory markers following Astragali radix supplementation is necessarily beneficial in the context of training-induced adaptation. The absence of between-group differences in muscle strength despite lower circulating inflammatory marker levels in the ASTRA group indicate that systemic attenuation of inflammation does not guarantee a corresponding improvement in functional or adaptive outcomes. This dissociation may partly reflect the physiological complexity of exercise-induced inflammation: TNF-α and IL-6, while associated with tissue stress at elevated concentrations, also serve as key signals for satellite cell recruitment, myonuclear accretion, and structural remodeling at the local muscle level [38,39]. Their systemic attenuation by Astragali radix supplementation may therefore not uniformly correspond to reduced intramuscular inflammatory activity and could in principle leave the adaptive signaling cascade largely intact—a distinction that circulating biomarker measurements alone cannot resolve. However, it is also true that, by focusing on maximal strength measures (MVIT and 1RM), our assessment may not have captured other functionally relevant dimensions of recovery, such as rate of force development, muscular endurance, or neuromuscular function, which could potentially be more sensitive to the anti-inflammatory effects of supplementation. Future studies incorporating muscle biopsy analyses, intramuscular molecular markers, and a broader battery of functional outcome measures would be necessary to establish whether the systemic anti-inflammatory effect of Astragali radix translates into meaningful improvements in muscle adaptation.

From an endocrine perspective, fluctuations in hormone levels, including testosterone and cortisol, have been commonly investigated following overload training [41,42]. The levels of cortisol were significantly reduced after the IT period in both groups, suggesting an impaired hypothalamic and pituitary response to exercise stimuli [43]. Additionally, the absence of changes in testosterone levels corroborates previous studies [41,42], which indicates that alterations in hormonal levels may not reliably serve as markers for detecting NFOR as hormonal changes can occur independently of performance outcomes [6]. In contrast, Astragali radix extract supplementation did not exert any positive effect on hormonal levels. This aligns with findings from another study in which a group of 16 rowers received a combination of Astragali radix and reishi for 30 days without showing any positive effect on testosterone or cortisol levels [44].

Finally, Astragali radix has been shown to activate intracellular signaling pathways that upregulate IGF-I during the early phase of muscle inflammation [44], which can promote muscle cell repair by activating satellite cells. Although we did not find significant differences between the two groups when examining the effect of Astragali radix extract on additional hormonal changes (IGF-1) and blood damage biomarkers (CPK and CRP) in the blood, it is possible that Astragali radix exerted intracellular effects on muscle repair and remodeling that were not captured by the circulating biomarkers measured in the present study. Future work incorporating muscle biopsies or more sensitive molecular markers would be needed to clarify this possibility. It should be acknowledged, however, that all biomarkers reported in the present study reflect systemic circulating concentrations, which only provide an indirect window into the intramuscular environment where training adaptations ultimately occur. The molecular events central to muscle remodeling—including satellite cell activation, intracellular anabolic signaling, and myofibrillar protein turnover—cannot be directly inferred from plasma cytokine or enzyme levels, and conclusions regarding the mechanistic basis of our findings must therefore be interpreted with appropriate caution.

### 4.3. Limitations and Future Directions

A potential limitation of the present study concerns the timing of the post-intervention testing. All neuromuscular and blood assessments were performed 24 h after the last training session; however, different physiological parameters may follow distinct recovery time courses, meaning that a single post-intervention time point may not capture the full trajectory of change for all variables. For instance, Yen and colleagues reported that blood biomarkers of muscle damage (CPK) were already elevated relative to the placebo group after just one day of intensified resistance training, whereas significant differences in muscle soreness assessed by the VAS only emerged after two days [17]. This suggests that functional outcomes, such as perceived soreness or force production, may require longer recovery periods to show measurable changes, while blood biomarkers can respond more rapidly. One mechanistic explanation is that enzymes such as CPK primarily reflect immediate sarcolemmal damage and Z-line disruption [45], whereas muscle soreness depends on secondary physiological processes that develop more slowly, including the inflammatory cascade, interstitial edema, and peripheral nociceptor sensitization [46,47]. Future studies should therefore consider incorporating multiple post-exercise assessment time points to better characterize the kinetics of both functional and biochemical recovery, and whether different recovery durations or recovery-management strategies influence the effectiveness of *Astragalus membranaceus* supplementation during intensified training periods.

A second consideration relates to the dosage of *Astragalus membranaceus* used in the present study relative to prior research. In animal models, significant effects have typically been reported at considerably higher doses compared to those used in human trials. For instance, Yeh and colleagues administered 0.615–3.075 g/kg/day to trained mice over six weeks, producing clear improvements in endurance capacity and reductions in post-exercise lactate and ammonia accumulation following acute exercise [48]. In human studies, however, meaningful benefits have been demonstrated at substantially lower doses. Latour and colleagues conducted a randomized controlled trial in which 500 mg of a standardized *Astragalus membranaceus* extract per day was sufficient to preserve lymphocyte counts and attenuate exercise-induced immunosuppression compared to a placebo, suggesting that moderate doses can effectively support immune function during intensive training [22]. Similarly, a recent randomized placebo-controlled study conducted in 90 adults aged 18–60 with functional knee pain over 28 days—with follow-ups at days 5, 14, and 28—found that 480 mg of Axtragyl^®^ per day, a dosage identical to that used in our study, significantly reduced knee pain and improved joint function, mobility, stair-climbing performance, and range of motion compared to the placebo [19]. Furthermore, Yen and colleagues demonstrated significant reductions in skeletal muscle damage biomarkers (serum CPK, LDH, and myoglobin) using a lower dosage than ours, specifically 4 mg of Astragalosides per day over 7 days [17].

Taken together, the available literature indicates that both the timing of assessment and dosage must be carefully considered when evaluating the effects of *Astragalus membranaceus* supplementation. While higher doses appear necessary to elicit effects in animal models, moderate doses can produce meaningful benefits in humans across a range of outcomes [17,19,22,48]. In light of this, the absence of significant effects on muscle soreness and certain blood biomarkers in the present study may be more plausibly attributable to the timing of assessment than to the dose administered. A further limitation of the present study is that participants were recruited from a single university and therefore represented a relatively homogeneous sample. As such, the external validity of the findings may be limited, and caution is warranted when generalizing these results to populations with different demographic, cultural, and educational backgrounds. Finally, it should be acknowledged that the range of motion was based on the participant’s subjective perceived discomfort, which may have influenced measurement consistency and between-subject comparability. Future studies incorporating extended follow-up periods and multiple post-intervention time points are warranted to more comprehensively evaluate the time course of these potential therapeutic benefits.

## 5. Conclusions

In conclusion, *Astragalus membranaceus* hydroalcoholic root extract supplementation did not significantly enhance muscle strength or mitigate force loss following an intensified strength training period. However, Astragali radix extract supplementation favorably modulated several inflammatory biomarkers, demonstrating an anti-inflammatory effect that may benefit athletes during periods of high training load. Overall, these results suggest that Astragali radix may have a beneficial role in attenuating inflammation in athletes exposed to high training loads during their preparation period.

## Figures and Tables

**Table 1:** Schematic representation of the strength training program designed for participants over a 10-week period, divided into a regular training block (RT) and an intensified training block (IT), which lasted 2 weeks, during which, athletes performed strength training sessions six times per week.

RT Period							IT Period	
Exercise	Weeks 1, 2	Workload (% 1RM)	Weeks 3, 4, 5	Workload (% 1RM)	Weeks 6, 7, 8	Workload (% 1RM)	Weeks 9, 10	Workload (% 1RM)
Back Squat	3 × 12	70%	3 × 10	75%	3 × 8	80%	-	
Leg Press	3 × 12	70%	3 × 10	75%	3 × 8	80%	10 × 1	100%
Single Leg Extension	3 × 12	70%	3 × 10	75%	3 × 8	80%	10 × 1	100%
Single Leg Curl	3 × 12	70%	3 × 10	75%	3 × 8	80%	-	
Stand Calf Raise	3 × 12	70%	3 × 10	75%	3 × 8	80%	-	

**Table 2:** Baseline characteristic of the subjects included in the group supplemented with Astragali radix extract (ASTRA) or control group (PLA).

	ASTRA	PLA
**Muscle strength**		
MVIT (Nm)	158 ± 75	153 ± 68
1RM leg press (Kg)	160 ± 55	145 ± 51
1RM leg extension (Kg)	31 ± 12	31 ± 12
**Knee joint mobility**		
ROM (degree°)	104 ± 6	106 ± 6
**Muscle soreness**		
VAS (1–100)	4.0 ± 6	1.3 ± 2
**Blood biomarkers**		
CPK (u/L)	137.6 ± 99	105.7 ± 72
LDH (u/L)	186.7 ± 43	186.7 ± 29
Mb (μg/dL)	27.9 ± 11	24.5 ± 10
IGF-1(ng/mL)	227.0 ± 72	211.5 ± 55
IL-6 (pg/mL)	1.199 ± 0.1	1.208 ± 0.1
TNF-α (pg/mL)	1.054 ± 0.1	1.051 ± 0.3
C (μg/dL)	13.4 ± 2.3	12.2 ± 4.5
T (nmol/L)	16.5 ± 18.0	16.6 ± 15.5

Values are given as mean ± SD. MVIT: maximal voluntary isometric contraction torque; 1RM leg press (Kg): one repetition maximum on leg press; 1RM leg extension (Kg): one repetition maximum on leg extension. ROM: range of motion; VAS (1–100): visual analog scale ranging from 1 to 100. CPK: creatine phosphokinase; LDH: lactate dehydrogenase; Mb: myoglobin; IGF-1: insulin growth factor-1; IL-6: interleukin-6; TNF-α: tumor necrosis factor-alpha; C: cortisol; T: testosterone. No differences were reported for any of the investigated variables.

## Data Availability

The raw data supporting the conclusions of this article will be made available by the authors on request.

## Abbreviations

ASTRA: Astragali radix supplementation group; PLA: placebo group; RT: resistance training; IT: intensified training; BAS: baseline; MVIT: Maximal Voluntary Isometric Torque; 1RM: one repetition maximum; ROM: Range of Motion; VAS: visual analog scale; CPK: creatine phosphokinase; LDH: lactate dehydrogenase; Mb: myoglobin; IGF-1: insulin-like growth factor 1; IL-6: interleukin 6; TNF-α: tumor necrosis factor alpha; ELISA: enzyme-linked immunosorbent assay; OTS: overtraining syndrome; NFOR: non-functional overreaching; IPAQ-SF: International Physical Activity Questionnaire—Short Form; METs: metabolic equivalents; CRP: C-reactive protein; IL2, IL4, IL10: interleukin 2, 4, 10; C: cortisol; T: testosterone.
